# Sensitivity of the Spatial Distribution of Fixations to Variations in the Type of Task Demand and Its Relation to Visual Entropy

**DOI:** 10.3389/fnhum.2021.642535

**Published:** 2021-06-08

**Authors:** Piero Maggi, Francesco Di Nocera

**Affiliations:** Department of Psychology, Sapienza University of Rome, Rome, Italy

**Keywords:** mental workload, eye-tracking, scanpath, fixations, entropy

## Abstract

Ocular activity is known to be sensitive to variations in mental workload, and recent studies have successfully related the distribution of eye fixations to the mental load. This study aimed to verify the effectiveness of the spatial distribution of fixations as a measure of mental workload and its sensitivity to different types of demands imposed by the task: mental, temporal, and physical. To test the research hypothesis, two experimental studies were run: Experiment 1 evaluated the sensitivity of an index of spatial distribution (Nearest Neighbor Index; NNI) to changes in workload. A sample of 30 participants participated in a within-subject design with different types of task demands (mental, temporal, physical) applied to Tetris game; Experiment 2 investigated the accuracy of the index through the analysis of 1-min epochs during the execution of a visual-spatial task (the “spot the differences” puzzle game). Additionally, NNI was compared to a better-known ocular mental workload index, the entropy rate. The data analysis showed a relation between the NNI and the different workload levels imposed by the tasks. In particular: Experiment 1 demonstrated that increased difficulty, due to higher temporal demand, led to a more dispersed pattern with respect to the baseline, whereas the mental demand led to a more grouped pattern of fixations with respect to the baseline; Experiment 2 indicated that the entropy rate and the NNI show a similar pattern over time, indicating high mental workload after the first minute of activity. That suggests that NNI highlights the greater presence of fixation groups and, accordingly, the entropy indicates a more regular and orderly scanpath. Both indices are sensitive to changes in workload and they seem to anticipate the drop in performance. However, the entropy rate is limited by the use of the areas of interest, making it impossible to apply it in dynamic contexts. Conversely, NNI works with the entire scanpath and it shows sensitivity to different types of task demands. These results confirm the NNI as a measure applicable to different contexts and its potential use as a trigger in adaptive systems implemented in high-risk settings, such as control rooms and transportation systems.

## Introduction

In high-risk environments characterized by highly dynamic, unpredictable, and uncertain events, many visual elements displayed in the complex control interfaces (e.g., monitoring sensors and warning indicators) tax operator’s attention causing cognitive overload. The restricted attentional capacity of the human being constitutes a well-known “bottleneck” that has been the object of many studies on human information processing (Marois and Ivanoff, 2005; Wolfe et al., 2006; Tombu et al., 2011) and on operator functional state, that is “the intrinsic relationship between human task performance and the background state of the individual” (Hockey et al., 2003). The topic is relevant in those environments where the presence of the human operator is essential for the management and the evaluation of unpredictable critical events. Indeed, it is important that the operator is always in the best psychophysical condition to reduce the risk of errors and accidents. The main objective of studies on human performance and information processing is to reduce the possibility of cognitive overload as much as possible. In the early 90s, Wickens (1992) highlighted the importance of achieving the highest compatibility between the operator’s capabilities and the characteristics of the surrounding environment. A mismatch between the machine and the operator can lead to a deterioration of performance and an increase in the workload (Hockey, 1986; Gaillard and Wientjes, 1994). To avoid this, finding real-time indexes of the operator functional state has become crucial. Also, this should be accomplished by using effective and non-intrusive tools to be applied in high-risk environments.

### Psychophysiological-Indexes as Mental Workload

Several psychophysiological measures have been studied for identifying new real–time indicators of operator functional state. Mehler et al. (2009) conducted a research study on a sample of 121 participants with the aim of examining the sensitivity of parameters such as heart rate variability, skin conductance, and respiratory rate as continuous measures of workload in a simulated driving environment. The analysis showed a significant effect of the difficulty level in all the psychophysiological parameters. A more recent study (Pakarinen et al., 2018) examined the relationship between the mental workload and the response to the physiological stress of nuclear power plant operators, who were assigned the task of managing the simulation of a large–scale accident through the control room. Records of heart rate and heart rate variability (respectively) were used to measure stress on a sample of 22 volunteer operators. The results showed a relation between the psychophysiological measures and the increase in workload experienced during a high accident risk scenario. In addition, these findings confirmed the data from self-report measures (NASA-TLX) and corroborated previous research results (Bernardi et al., 2000; Hwang et al., 2008; Reimer and Mehler, 2011). More specifically, many studies in the literature have investigated the relationship between neurophysiological measures and mental workload, such as electroencephalography (EEG; Brookings et al., 1996; Gevins and Smith, 2003; Borghini et al., 2014), functional Near-InfraRed Spectroscopy (fNIRS), and functional Magnetic Resonance Imaging (fMRI; Gabbard et al., 2017; Liu et al., 2017; Ranchet et al., 2017). In a study by Aricò et al. (2016), a workload index based on EEG measurements was used as a trigger in an adaptive automation system implemented in a realistic Air Traffic Control Environment.

### Ocular Metrics as Mental Workload Indicators

The need to apply sensors on the operator’s body during the execution of a task poses a major limitation for the use of the previously mentioned psychophysiological parameters (i.e., skin conductance, respiratory rate, and heart rate variability). Indeed, the invasiveness of these tools does not facilitate their implementation in the operative setting. Among the real–time measures of mental workload, ocular activity is the least invasive and most promising (Ellis, 2009; Singh and Singh, 2012; McIntire et al., 2014). Eye trackers can measure different eye parameters including the direction of gaze, changes in pupil diameter, and eye-blinks. Several metrics can be computed (e.g., number and duration of fixations, saccades amplitude), to obtain a graphic representation of the individual behavior. Moreover, it is important to underline that research in this field has continued to evolve thanks to continuous technological innovation, which has led to increasingly advanced, less intrusive, and more accurate instruments for monitoring eye activity (Wang et al., 2017).

### Pupil Diameter

Since the seminal studies by Beatty and Kahneman (1966), several authors have shown that pupil dilation may be related to cognitive processing and to the mental effort required to perform a given task (Othman and Romli, 2016; Kosch et al., 2018; van der Wel and van Steenbergen, 2018). This relation has been analyzed in various tasks including short–term memory (Peavler, 1974) and visual search tasks (Porter et al., 2007), but also in air traffic control (Hilburn et al., 1997) and driving (Rezaei and Klette, 2011). Just and Carpenter (1993) identified changes in pupil diameter when understanding single sentences with different degrees of difficulty. Iqbal et al. (2004) confirmed a correlation between pupil variation and mental workload when participants were asked to perform various tasks including text comprehension, mathematical reasoning, target stimulus research, and object manipulation.

The pupil diameter has been reported to be very promising as a measure of mental workload. However, an important limitation of this measure is the difficulty of keeping constant the brightness of the environment in which the task is performed. The amount of light reaching the eye causes rapid changes in pupil diameter and this can limit the benefits of this metric in working environments. In fact, unlike controlled laboratory settings, the brightness of the working environment (e.g., the brightness of the displays or the room) is variable. Changes in pupil diameter may be due partly to physical components in the environment and partly to workload, making it difficult to isolate a valid and reliable measure of cognitive effort.

### Fixations and Saccades

The highest visual acuity is limited to the foveal area and fixations represent the point in time and space of focus, whereas saccades are eye movements that are necessary to bring image portions on the fovea, therefore vision is obtained by moving the focus from one point (fixation) to another. These two elements together (and their properties like frequency, duration, amplitude) make up the entire visual search strategy adopted by the individual to examine a scene.

Since the seminal studies by Yarbus (1967), many authors have investigated visual exploration strategies showing a systematic relationship between fixation duration and saccadic amplitude (Antes, 1974; Findlay and Gilchrist, 2003; Pannasch et al., 2008). These results are also explained as an adaptation in the exploration strategy used to perform a task (Underwood et al., 2011). The relationship between fixation duration and saccadic amplitude is of particular interest for its possible diagnostic value (Velichkovsky et al., 2000, 2002; Unema et al., 2005).

### Scanpath Analysis

Regarding scanpath, namely the sequence of fixations and saccades, few studies have investigated its features in relation to other factors, such as mental workload. The topography of the visual scanning, as well as its dynamics, was quantitatively approached in two studies by Tole et al. (1983) and Harris et al. (1986) who suggested to using the entropy rate of the visual scanning for discriminating between different levels of mental workload. Their results suggested that the scanpath tends to be cluttered and random when the workload is low. Instead, it would become regular and predictable as the demand increases. Although very appealing, entropy has seldom been used as a measure of workload and therefore its properties have not been properly tested. Moreover, entropy is limited by the need to rely on predefined Areas of Interest (AOIs) in order to compute transitions between them: in many operational settings visual scanning happens outside specific AOIs, or the AOIs change dynamically. To overcome this limit, Di Nocera et al. (2007) introduced an alternative approach based on the spatial distribution analysis of fixations using the Nearest Neighbor Index (NNI). The spatial distribution showed sensitivity to changes in mental workload. Studies on its functional significance suggest that scanpath may be more scattered when the temporal demand increases (i.e., time pressure), whereas the visuo-spatial demand would be responsible for higher concentration of fixations in specific areas (Camilli et al., 2008a). The clustering of fixations in specific areas is similar to the concept of an ordered scanpath that was underlain by the entropy rate. Indeed, entropy is based on transitions between AOIs and it was applied to scenarios within which changes in the task load were due to changes in the visuospatial demand. However, the two metrics have never been compared. The analytical details of the two approaches will be described in the following sections.

#### Entropy Rate

Entropy can be defined as a measure of the disorder found in any physical system and this concept was then applied by Tole et al. (1983) to eye movements. When the individual looks at all the quadrants in the scene and crosses all the potential combinations of stimuli with a stable frequency, the entropy will increase. Instead, the entropy value will be lower when the individual focuses attention on a narrower range of possible areas of interest. That happens because the frequency of transitions from one area to another decreases. A regular and systematic visual exploration strategy is shown in a condition of low entropy, which corresponds to a more orderly passage to other areas. The principal benefit of this analysis is the possibility to “summarize” the visual strategy using a single value. The first step in estimating the amount of entropy is to identify the areas of interest in the visual field, and then computing the proportion of time taken by the participant to look at each of these areas:

$$Entropy\;rate=\sum_{i=1}^{D}\left[(E/E\_\max)/DT_{i}\right]$$

$$E=-\sum_{i=1}^{D}P_{i}\log_{2}P_{i}$$

where *E* represents the value of the observed average entropy, *E_max_* is the maximum entropy value computed from the total number of AOIs in the scene (it constitutes the entropy value when all AOIs are accessed with the same probability), *P_i_* represents the probability that the sequence *i* occurs, *DT_i_* is the average duration of fixation for the *i*-th sequence when the individual is visually exploring the scene, and D expresses the number of the distinct sequences in the scanpath. The index is indicated in bits/second.

#### Nearest Neighbor Index

The NNI provides data on the distribution of points in space. The average distance between the fixations collected during the execution of a task and the average distance between the fixations expected in a random distribution are taken into account in the application of the NNI to eye movements. The result is represented by a single value where one indicates that the empirical and the random distribution are not different; values above one indicate dispersion, while values below one show clustering. The index can be computed for small epochs if sufficient fixations are available (about 50 as a rule of thumb) and then analyzed as a time series, therefore offering information on the temporal variations of distribution of fixation points. A methodological study (Camilli et al., 2007) supports the validity of this algorithm as a measure of mental workload, highlighting the consistency of the index with subjective and psychophysiological measures. To estimate the index it is first necessary to calculate the Nearest Neighbor distance or *d(NN)*:

$$d(NN)=\sum_{i=1}^{N}\left[\min\frac{\left(dij\right)}{N}\right],\;1\leq j\leq N,\;j\neq 1$$

where min (*dij*) represents the distance existing between each point *i* and the nearest point *j* (with the *j* value between 1 and *N* and different from *i*), and *N* corresponds to the number of points in the distribution. The next step is calculating the average random distance or *d(ran)* to obtain the second element of the equation; this value would represent the value of *d(NN)*, supposing that the distribution of the points was totally random:

$$d(ran)=0.5\sqrt{\frac{A}{N}}$$

where A indicates the polygon area delineated by the most extreme fixations and N represents the number of points. The NNI value is then calculated by dividing the Nearest Neighbor Index distance, *d(NN)*, by the average random distance, *d(ran)*:

$$NNI=\frac{d(NN)}{d(ran)}$$

## Experiment 1

The objective of this experiment was to test the sensitivity and diagnosticity (see Wierwille and Eggemeier, 1993) of the NNI, that is how changes in the visual exploration strategy due to different workload levels and different types of demand imposed by the task are captured by the distribution of fixations. This aspect has been previously approached by Camilli et al. (2008b) in a between-subject design, comparing the effects of the mental and temporal demands on the distribution of fixations.

### Tools and Software

#### Experimental Software Development

The Tetris game used in this study was coded using Javascript and Google script. The gaming area consisted of 300 cells deployed on a grid of 15 columns by 20 rows. Each tetromino (piece) was randomly extracted from a pool composed of seven different tetromino types and it descended at a constant speed. With the aim of creating three experimental conditions, specific variables have been modified to induce a different type of task demand. In Condition 1, the speed of falling pieces has been manipulated to generate time pressure (temporal demand); in Condition 2, the direction of pieces has been reversed to increase mental demand (each piece appears in the lower part of the game area and then rises to the top); in Condition 3, the interaction with pieces was occasionally blocked, therefore forcing the user to press the control keys several times to move the pieces (physical demand).

The manipulations were coherent with the NASA-TLX definition of mental, temporal, and physical demand. Mental demand: “How much mental and perceptual activity was required? Was the task easy or demanding, simple or complex?” Temporal demand: “How much time pressure did you feel due to the pace at which the tasks or task elements occurred? Was the pace slow or rapid?” Physical demand: “How much physical activity was required? Was the task easy or demanding, slow or brisk, slack or strenuous, restful or laborious?” Therefore, we consider the visuospatial demand imposed here as an expression of the mental demand.

In our version of Tetris, the “game-over” consisted of the exhaustion of the playing area given by the excessive accumulation of pieces but did not represent the end of the game. When the event occurs, the program automatically resets the entire area deleting all the accumulated pieces and allowing the user to continue the game until the end of the experiment. The number of pieces accommodated and the number of completed lines were used as performance measures. The number and shape of the pieces, the size of the playing area, and the difficulty between levels were based on the original version of the Tetris.

#### Ocular Activity Recordings

The Gazepoint GP3HD eye-tracking system was used to record ocular activity. This system allows the researcher to collect ocular data without using invasive and/or uncomfortable head-mounted instruments. Gazepoint, the eye tracker manufacturer, claims accuracy within 0.5–1.0 degrees and reads data at a rate of 150 Hz. The eye tracker was calibrated using the default 9-point calibration test using Gazepoint’s included software.

### Participants

Thirty university students (19 women and 11 males, *M* = 25 years old, *SD* = 3.6) volunteered and participated in the experiment. All participants had a normal or corrected-to-normal vision and were naïve as to the aims of the experiment. This research study was completed with the tenets of the Declaration of Helsinki and was approved by the Institutional Review Board of the Department of Psychology, Sapienza University of Rome. Informed consent was obtained from each participant. Participants received a €20.00 worth bookstore gift card.

### Procedure

Participants were tested in a within-subject design in which the same task was manipulated-in three different sessions- for manipulating the mental, the temporal, and the physical demand. Participants played a custom-coded version of the Tetris game, a commonly known tile-matching puzzle videogame successfully used in a variety of studies (e.g., Trimmel and Huber, 1998). For experimental purposes, the game restarted from a blank screen each time the stack of Tetriminos reached the top of the gaming area and no new Tetriminos were able to enter. This condition commonly denotes the end of the game, whereas in this experiment it was scored as a loss (performance measure). Participants were instructed to gain as many points as possible (i.e., complete lines and avoid losses).

#### Training Session

Before the experimental session started, each participant performed a training session, whose scope was to familiarize the participants with the experimental setting. To this aim, each participant played the Tetris game starting from a low difficulty level and moving on to Baseline, TD, MD, and PD conditions. The training had a 5-min duration and did not include the evaluation of the participants’ performance level in this phase.

The scheme of the training session is reported below:

• One minute of gameplay at Level 1 (drop speed: 1,250 ms per block), with the aim of verifying the correct understanding of the game rules and allowing the participant to familiarize themselves with the use of directional keys.
• Baseline condition: 1 min, configured at level 6 (drop speed: 208 ms per block). It was used to acquire the baseline for the experimental session.
• TD condition: 1 min, set at level 8 (drop speed: 156 ms per block).
• MD condition: 1 min, during which the entire playing area was rotated by 180° and each Tetromino appeared on the bottom side and went up, accumulating on the top of the gaming area.
• PD condition: 1 min, in which the participant needed to press the directional keys repeatedly to move the piece quickly in the chosen direction (instead of keeping the key pressed).

#### Experimental Session

After the calibration of the eye-tracker, participants were instructed to play the game earning as many points as possible (i.e., complete lines and avoid losses). Each condition lasted 10 min and the order of presentation was randomized across participants. After completing each condition (Baseline vs. TD vs. MD vs. PD), participants were requested to fill in the NASA-TLX (Hart and Staveland, 1988).

### Data Analysis and Results

#### Performance Data

**Figure 1:**
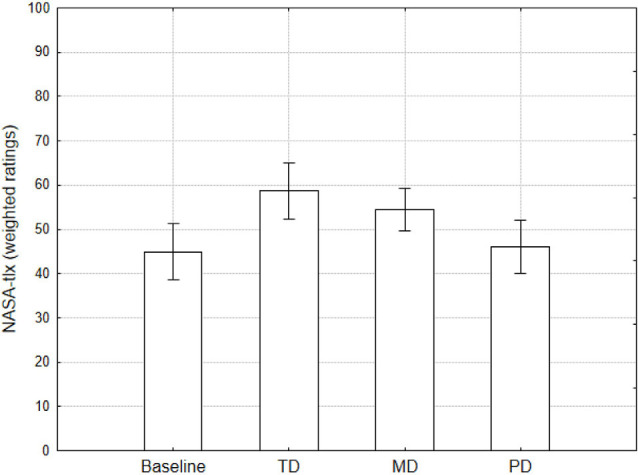
The performance index shows the overall strategy used by participants. It is the ratio of the number of completed lines to the number of pieces that appeared during the game. Values close to 1 mean an optimal game with a high number of lines completed.

A performance index was computed based on the number of lines completed in relation to the maximum number of lines that could be completed. The maximum value is obtained by the total number of Tetrominoes that the participant managed in each condition (For example, with 60 pieces it was possible to complete a total of 16 lines if managed in an optimal way). The index goes from 0 to 1, where 1 means that the player has obtained the maximum achievable score. The performance index was used as a dependent variable in a repeated measures ANOVA design, using Condition (Baseline vs. TD vs. MD vs. PD) as repeated factor. Results showed a main effect of the condition (*F*_(3,87)_ = 15.95, *p* < 0.001). The faster (TD) and Reversal conditions (MD) were associated with the worse performance with respect to the baseline (Figure 1).

#### Subjective Measure

**Figure 2:**
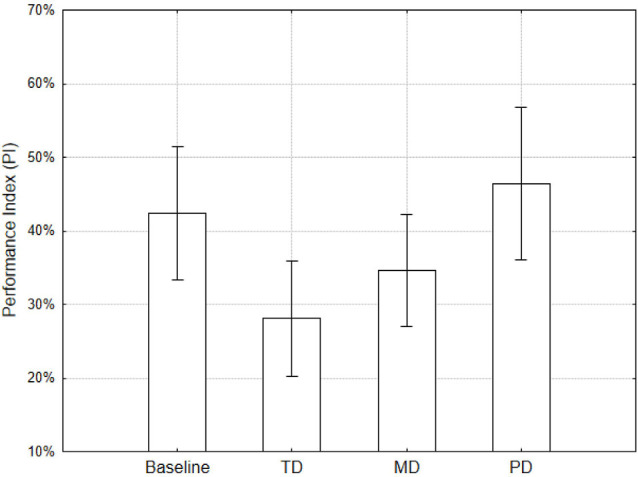
NASA-TLX values (weighted scores) separately for the conditions. Error bars denote 0.95 confidence intervals.

**Figure 3:**
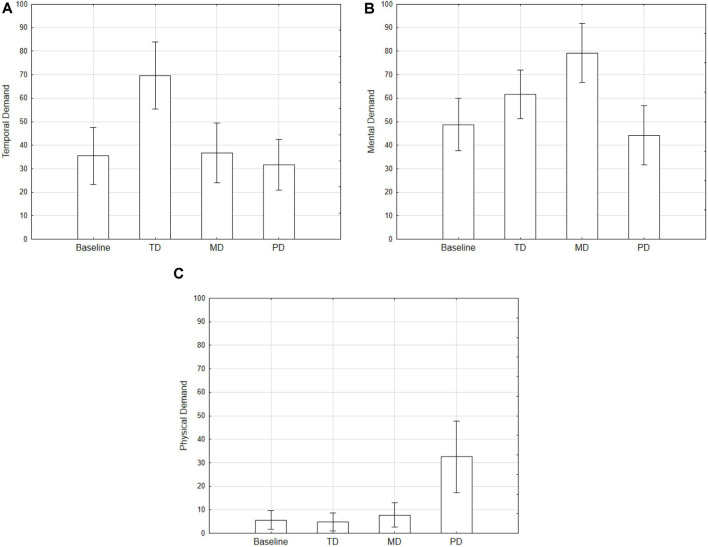
NASA-TLX subscales values [Temporal **(A)**, Mental **(B)**, and Physical **(C)** demand] separately for the conditions. Error bars denote 0.95 confidence intervals.

NASA-TLX weighted ratings were used as dependent variables in a repeated measures ANOVA design using Condition as repeated factor. Results showed a main effect of Condition (*F*_(3,87)_ = 12.3, *p* < 0.001; Figure 2), consistent with those obtained for the performance index. Although analyses on the single items are questionable from a statistical standpoint, it is worth noting that TD, MD, and PD conditions showed higher values for temporal, mental, and physical demand scales respectively (Figure 3). These results show that the manipulations made with the Tetris have indeed taxed specific aspects or resources.

#### Eye Tracking Metrics

##### Number and Duration of Fixations

**Figure 4:**
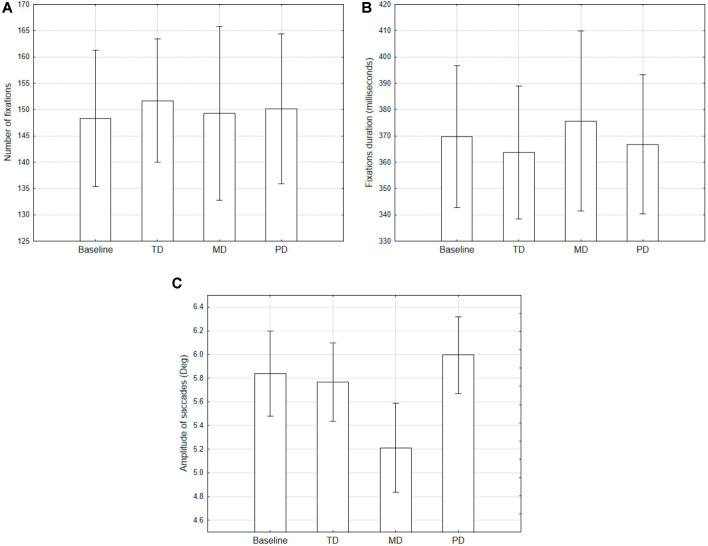
Averaged number **(A)** and duration **(B)** of fixations, and amplitude of saccades **(C)**, for the conditions compared with the baseline. Error bars denote 0.95 confidence intervals.

The number and duration of fixations were computed on epochs of 1 min for each participant and then averaged. One participant was excluded from the analysis due to the low quality of recorded eye movements. Averaged number and duration of fixations were used as dependent variables in a repeated measures ANOVA design using Condition as the repeated factor. No significant differences between conditions were found (Figures 4A,B panels).

##### Amplitude of Saccades

The amplitude of saccades was computed on epochs of 1 min for each participant and then averaged. The averaged amplitude of saccades was used as dependent variable in a repeated measures ANOVA design using conditions as a repeated factor (Figure 4C). Results showed a main effect of condition (*F*_(3,84)_ = 12.84, *p* < 0.001).

##### Nearest Neighbor Index

**Figure 5:**
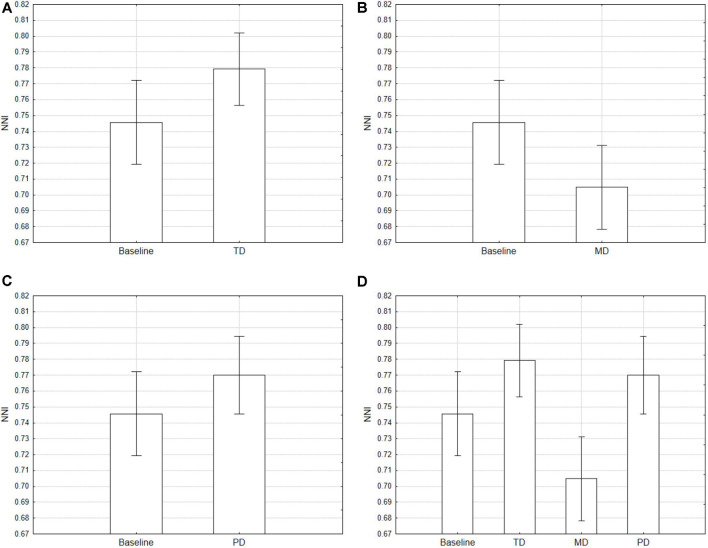
Average Nearest Neighbor Index (NNI) for the conditions compared with the baseline separately. Error bars denote 0.95 confidence intervals. Baseline *vs*. TD **(A)**; Baseline *vs*. MD **(B)**; Baseline *vs*. PD **(C)**; all condition **(D)**.

The NNI was computed on epochs of 1 min for each participant and then averaged. One participant was excluded from the data analysis due to the low quality of recorded eye movements. Averaged NNI values were used as the dependent variable in repeated measures ANOVA using conditions as the repeated factor. Results showed a main effect of Condition (*F*_(3,84)_ = 12.31, *p* < 0.001). TD condition showed higher NNI values (i.e., a more dispersed distribution of fixations) than the baseline (Figure 5A), while in the MD condition NNI values were the lowest (Figure 5B).

### Discussion

This first study aimed at investigating how the visual exploration strategy changes both along with the taskload and with the type of task demand. The results showed an increase in the NASA-TLX values of the single subscales (mental demand, physical demand, and temporal demand) matching the respective manipulation. Overall, we observed a greater workload in the MD and TD conditions compared to the control and PD conditions. The latter has shown higher values in the corresponding NASA-TLX scale, but the manipulation of the physical demand did not affect the overall self-reported workload. Finally, and more important to our aims, the analysis of the fixations pattern showed high clustering when the taskload increment was obtained by changing the mental (visuospatial) demand, and low clustering when it was obtained by changing the temporal demand.

## Experiment 2

The entropy-based analysis of the scanpath and the spatial distribution of fixations points are reported to be good indices of mental workload. However, they have never been directly compared. The aim of this second study is to perform such a comparison. A preliminary account on this experiment was presented at the H-WORKLOAD 2019 workshop (Maggi et al., 2019).

### Participants

The experiment involved 14 university students (nine women and six males, mean age = 24 years, *SD* = 2.6) who participated on a voluntary basis. All participants had a normal or corrected-to-normal vision and were naïve as to the aims of the experiment. This study was compliant with the principles of the Declaration of Helsinki and the protocol was approved by the Institutional Review Board of the Department of Psychology, Sapienza University of Rome. Each participant provided informed consent.

### Tools and Software

#### Stimuli

**Figure 6:**
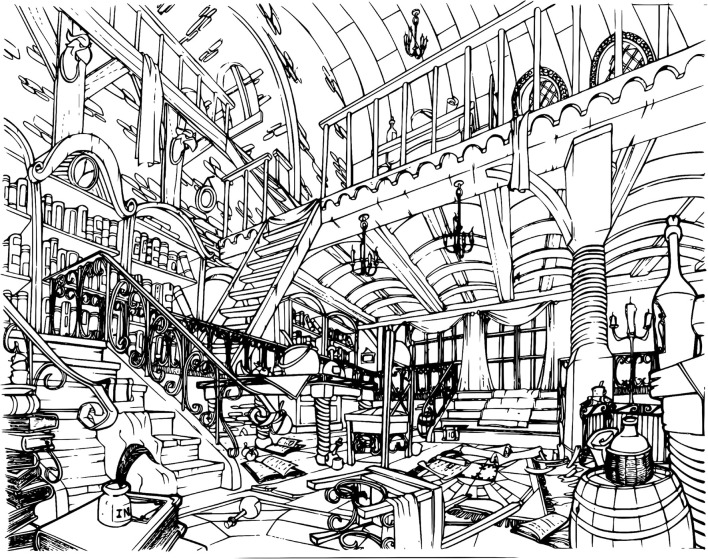
Left panel. Artwork by Benoit Tranchet (reproduced with permission).

**Figure 7:**
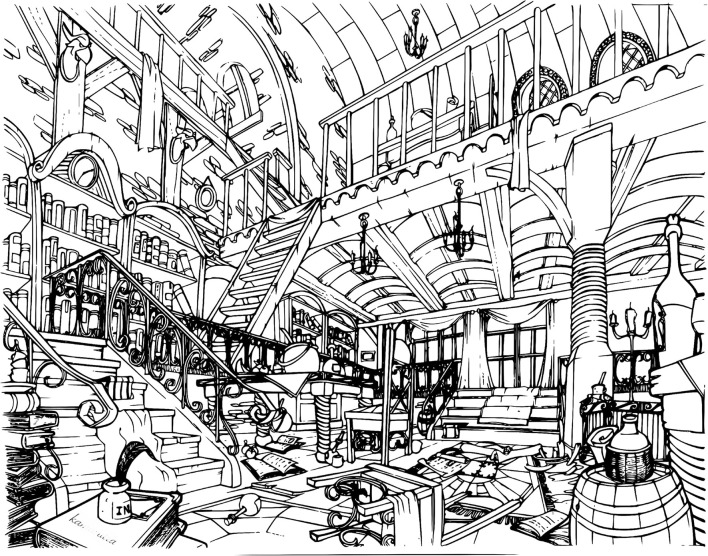
Right panel. Modified version of the original artwork with 35 differences.

In order to induce high visual-spatial demand and to assess how that affects visual search, a single pair of black and white pictures (Figures 6, 7) was used. Pictures were rich in details so that the numerous elements would engage participants in a long visual exploration session. The size of each picture was 9.8 × 5.5 inches and both of them featured 35 subtle differences but were otherwise identical. The two images were aligned horizontally and in fullscreen mode on a 27′′ display.

#### Eye Movements Recording

Prior recording, participants performed a 9-point calibration and then their eye movements were recorded through the Pupil Labs system with binocular 120 Hz Eye Tracking Camera (Pupil Labs GmbH, Germany).

### Procedure

The experiment was conducted in a dark room and participants were seated at approximately 2 ft. from a computer screen. During the task, they had to find as many differences as they could between the two images in a 24-min session. They were requested to click with the mouse on each difference they identified. The differences found were highlighted with a circle throughout the session. Participants were also asked to provide a subjective evaluation of mental workload on a 2-min schedule [Instantaneous Self-Assessment (ISA): Tattersall and Foord, 1996].

### Data Analysis and Results

#### Performance and Self-reporting Measures

**Figure 8:**
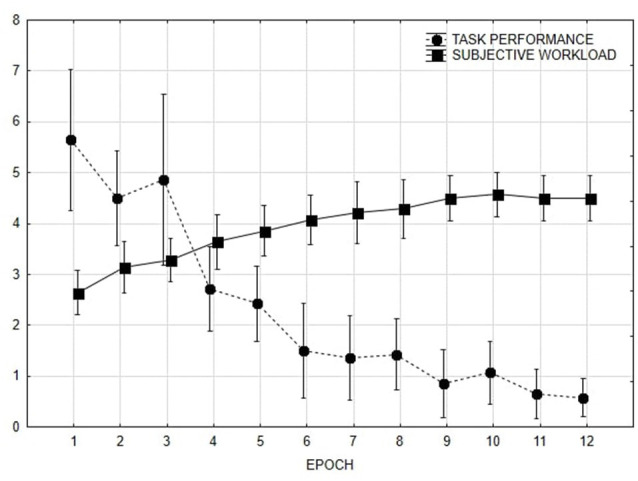
Task performance (number of differences found) and subjective workload (ratings from 1 to 5) along time. Error bars denote 0.95 confidence intervals.

The whole activity was split into 12 periods of 2 min each in order to match performance and subjective evaluations. The number of differences identified by each subject in each epoch was used as a performance indicator. The number of differences identified and the ISA scores were used as dependent variables in two repeated measures ANOVA designs using Epoch as repeated factor. A main effect of Epoch was found both on the number of differences (*F*_(11,143)_ = 16.52, *p* < 0.001; Figure 8) and the ISA scores (*F*_(11,143)_ = 15.50, *p* < 0.001; Figure 8). Plots reveal Duncan *post hoc* testing revealed asymptotic pattern for both the performance measure and the workload estimates starting from the 12th min.

#### Analysis of Eye-Tracking Data

##### Nearest Neighbor Index

**Figure 9:**
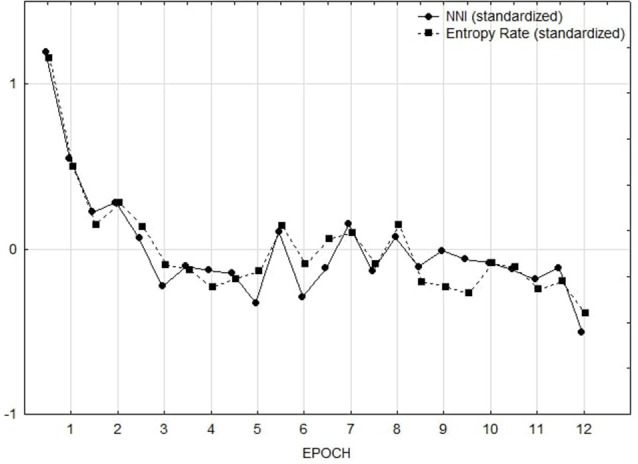
NNI and Entropy rate by epoch. Measures have been standardized (z-scores) to plot them together.

For each participant, the NNI was calculated taking into account 1-min epochs (Di Nocera et al., 2015). Average NNI values were used as dependent variables in a repeated measures ANOVA design using Epoch as repeated factor. A main effect of the Epoch was found (*F*_(11,143)_ = 4.41, *p* < 0.001; Figure 9). Duncan *post hoc* testing showed that the visual strategy applied in the first 2 min significantly differs from all other periods.

##### Entropy Rate

The whole visual area has been divided into two Areas of Interest (AOI), namely the two images displayed. For each minute, the maximum number and duration of fixations made on each AOI were assessed. For these AOIs, the entropy rate has been adopted as a measure of scan randomness (Tole et al., 1983). The entropy rate (H-rate) is expressed in units of bit/s (i.e., the information given by each observation, assessed in bits over seconds). A random pattern is represented by a high H-rate. In this study, all the scanpaths performed by the participants were used to compute the entropy rate. The entropy rates (H_rate) of the sequences of one length for the two images used were computed as a measure of the randomness of the scan. Average H_rate values were used as dependent variables in a repeated measures ANOVA design using the epoch as repeated factor. A main effect of time (*F*_(11.143)_ = 3.69, *p* < 0.001) was found. Duncan *post hoc* testing showed a steady pattern in the first 2 min of visual exploration, consistently with that obtained with the NNI.

### Discussion

This second study aimed at comparing two scanpath analysis methods that have been previously reported to be sensitive to changes in the taskload: Entropy rate and Nearest Neighbor Index. Results showed an overall increase of difficulty after the first few minutes of the task. The entropy rate confirms the presence of a less random and more stereotyped pattern starting from the second minute of recording. A similar trend was found for the NNI. The average NNI values in the first 2 min of activity were significantly higher than in the following epochs, therefore showing a change towards fixations grouping as the taskload increased. This study was designed to evaluate the potential of these two measures under the effect of increasing visual-spatial demand. The results showed the same trend, therefore confirming that the two indices are sensitive to changes in the visuospatial demand. However, unlike the entropy rate, the NNI is also suitable for estimating changes due to the temporal demand (see Study 1). This is an aspect that could not be accommodated by the entropy rate, which is based on the transitions between AOIs, hence it is completely based on the visuospatial performance.

## General Discussion and Conclusions

This article reported a set of two studies designed to shed light on the relationship between mental workload and ocular scanning. This topic has been covered in the Human Factors/Ergonomics literature by using different approaches, but a complete understanding of that relationship is still a long way off. Previous studies of our laboratory have explored the opportunity to use the distribution of eye fixations as an indicator of mental workload. The Nearest Neighbor Index, a spatial statistics providing information about the distribution of points into a two-dimensional space, was found to be sensitive to variations in mental workload. However, results obtained using the NNI were apparently different from those obtained in accredited studies using scanning randomness or entropy for summarizing the scanpath, therefore questioning the value of this approach. Di Nocera and Bolia (2007) had initially speculated that the two processes respectively contribute to dispersion and grouping of the fixations: the temporal demand (that was manipulated in the NNI studies) and the visuospatial demand (that was manipulated in other studies, including those featuring entropy). That idea was partially tested by Camilli et al. (2008b) in a small between-subject study, but never deepened since then.

Indirect measures of mental workload (they all are) can be more or less sensitive to variations in the taskload imposed on the individual. Many of them can provide only a coarse distinction between taskload levels, others have been reported to be more fine-grained. Nonetheless, sensitivity to taskload variation is not the only important property of a successful indicator: sensitivity to different types of task demands is also important. Indeed, what we call mental workload (independently of its conceptualization) may be generated in response to changes in the taskload that may be due to changes in the visuospatial component of the task (i.e., the task becomes more demanding because the individual needs to look more, to find more, to discriminate more) or the taskload may be due to changes in the temporal component of the task (i.e., the task becomes faster, the interval between incoming stimuli becomes shorter, the time pressure for responding increases). The different types of demand are well represented by the NASA-TLX that features three scales named mental demand, temporal demand, and physical demand (while the other three scales represent the individual reaction in terms of performance, effort, and frustration).

The first study reported here was designed to test the diagnosticity of the NNI, that is how the fixations distribution varied not only along with the taskload but also with the type of task demand. The results showed high clustering when the taskload increment was obtained by changing the mental (visuospatial) demand, and low clustering when it was obtained by changing the temporal demand. The physical demand, instead, did not affect the scanpath, possibly because our manipulation of this dimension was not appropriate or because the ocular behavior is not sensitive to the manipulation of the physical component (see Figures 5C,D).

In the second study, instead, the NNI was directly compared to the entropy approach that is considered one of the most prominent techniques for studying the scanpath in the HF/E domain. Results showed an overall increase of difficulty after the first few minutes of performance that reflected in both measures of mental workload. After 2 min, the search task generated both a stereotyped dwell pattern (consistent with the entropy prediction) and fixations grouping (consistent with the fixations distribution prediction). In other words, the two indices were found to be both sensitive to changes in the visuospatial demand and the plots were highly overlapping. Such a result sorts out the issue of the differences found between the two indicators, showing how that exclusively depends on the type of demand imposed. Also, results demonstrated that a dispersed fixation pattern (or moderately grouped) is not equivalent to high randomness in visual exploration.

Of course, the studies reported here are not without limitations. As we have already reported one critical aspect is that related to the manipulation of the physical demand. Albeit results showed a significant increase in the subjective estimates of physical demand, the effect did not extend to the overall workload ratings nor to the analysis of the scanpath. Likely, the Tetris game involved minimal physical effort and the manipulation was not effective. To overcome this limitation, future studies could consider several options. One potential solution could be to manipulate the game controls producing frequent keypress failures in the high taskload condition. Alternatively, the keypress force could be manipulated in the high taskload condition to make the task more effortful.

The second study, instead, was designed with the validation of the NNI in mind. The “spot the difference” task was useful for providing a common ground to NNI and entropy using the type of task used in entropy studies (shifting from one AOI to another). Accordingly, only the sensitivity to the visuospatial demand was tested for both indicators while the sensitivity of the entropy measure to the temporal demand was not tested. Future studies should address also this aspect by keeping constant the visuospatial demand and increasing only the temporal demand. Nevertheless, this would be necessary only for sake of completeness, because the dependency on the AOIs is a strong limitation of the entropy approach, and the freedom offered by the NNI in the analysis of the ocular activity during the execution of any task is much more appealing.

In conclusion, the NNI is suitable for estimating changes due both to the temporal and the visuospatial demand, therefore showing diagnosticity, which is an important property for an effective indicator of mental workload.

## Data Availability Statement

The raw data supporting the conclusions of this article will be made available by the authors, without undue reservation.

## Ethics Statement

The studies involving human participants were reviewed and approved by Department of Psychology, Sapienza University of Rome. The participants provided their written informed consent to participate in this study.

## Author Contributions

PM and FDN have contributed equally to this work. All authors contributed to the article and approved the submitted version.

## Conflict of Interest

The authors declare that the research was conducted in the absence of any commercial or financial relationships that could be construed as a potential conflict of interest.
